# Ternary combination of irinotecan, fluorouracil-folinic acid and oxaliplatin: results on human colon cancer cell lines

**DOI:** 10.1054/bjoc.2000.1600

**Published:** 2001-02

**Authors:** J-L Fischel, P Rostagno, P Formento, A Dubreuil, M-C Etienne, G Milano

**Affiliations:** Oncopharmacology Unit, Centre Antoine Lacassagne, 33 Avenue de Valombrose, 06189 Nice Cedex 2, France

**Keywords:** irinotecan, fluorouracil, oxaliplatin, colon cancer cell lines, drug combination

## Abstract

A marked antitumour efficacy is currently obtained by oxaliplatin (LOHP)–fluorouracil (FU)–folinic acid (FA) combination and by CPT11–FU–FA combination. Logically, the triple association LOHP, CPT11 and FUFA will be soon tested in cancer patients. The aim of the present study was to compare two schedules combining SN38 (the active metabolite of CPT11, irinotecan) with FU–FA and LOHP. The two schedules differed by the SN38 position. The relative contribution of each drug in the resulting global cytotoxicity was evaluated. Two human colon cancer cell lines were used (WIDR and SW620 both p53 mutated). LOHP plus FA were applied for 2 h, just before a 48 h FU exposure. The SN38 sequence was applied for 24 h, starting either 48 h before LOHP-FA (schedule A), or just after LOHP-FA exposure (schedule B). Cytotoxicity was assessed by the 3-(4,5-demethylthiazol-2-yl)-2,5 diphenyltetrazolium bromide (MTT) test and drug interactions were analysed according to the Chou and Talalay method, based on the computation of a combination index (CI). The SN38 position significantly induces a shift from additivity-antagonism when SN38 was applied after LOHP, towards additivity-synergism when SN38 was applied first (*P* = 0.03). The relative contribution (RC) of each drug in the overall cytotoxicity of the triple combination was defined as the drug concentration giving 50% cell lethality (IC _50_) of the double association without that drug divided by the IC _50_ of the triple association. Whatever the SN38 position, the larger contribution was made by LOHP (median RC = 2.4) and the smaller by SN38 (median RC = 1.1). In addition, the contribution of FUFA was improved when SN38 was applied first (median RC = 2.2) as compared to the opposite schedule (median RC = 1.2). Results were in agreement between the two explored cell lines. The present data should be taken into account when establishing the rationale of future trials combining CPT11, LOHP and FU–FA. © 2001 Cancer Research Campaign http://www.bjcancer.com


British Journal of Cancer (2001) **84**(4), 579–585
© 2001 Cancer Research Campaign
doi: 10.1054/ bjoc.2000.1600, available online at http://www.idealibrary.com on IDEAL®

http://www.bjcancer.com

### Ternary combination of irinotecan, fluorouracil-folinic acid and oxaliplatin: results on human colon cancer cell lines

**J-L Fischel, P Rostagno, P Formento, A Dubreuil, M-C Etienne and G Milano**

Oncopharmacology Unit, Centre Antoine Lacassagne, 33 Avenue de Valombrose, 06189 Nice Cedex 2, France

**Summary** A marked antitumour efficacy is currently obtained by oxaliplatin (LOHP)–fluorouracil (FU)–folinic acid (FA) combination and by CPT11–FU–FA combination. Logically, the triple association LOHP, CPT11 and FUFA will be soon tested in cancer patients. The aim of the present study was to compare two schedules combining SN38 (the active metabolite of CPT11, irinotecan) with FU–FA and LOHP. The two schedules differed by the SN38 position. The relative contribution of each drug in the resulting global cytotoxicity was evaluated. Two human colon cancer cell lines were used (WIDR and SW620 both p53 mutated). LOHP plus FA were applied for 2 h, just before a 48 h FU exposure. The SN38 sequence was applied for 24 h, starting either 48 h before LOHP-FA (schedule A), or just after LOHP-FA exposure (schedule B). Cytotoxicity was assessed by the 3-(4,5-demethylthiazol-2-yl)-2,5 diphenyltetrazolium bromide (MTT) test and drug interactions were analysed according to the Chou and Talalay method, based on the computation of a combination index (CI). The SN38 position significantly induces a shift from additivity-antagonism when SN38 was applied after LOHP, towards additivity-synergism when SN38 was applied first ($P$ = 0.03). The relative contribution (RC) of each drug in the overall cytotoxicity of the triple combination was defined as the drug concentration giving 50% cell lethality ($IC_{50}$) of the double association without that drug divided by the $IC_{50}$ of the triple association. Whatever the SN38 position, the larger contribution was made by LOHP (median RC = 2.4) and the smaller by SN38 (median RC = 1.1). In addition, the contribution of FUFA was improved when SN38 was applied first (median RC = 2.2) as compared to the opposite schedule (median RC = 1.2). Results were in agreement between the two explored cell lines. The present data should be taken into account when establishing the rationale of future trials combining CPT11, LOHP and FU–FA. © 2001 Cancer Research Campaign htt://www.bjcancer.com

**Keywords:** irinotecan; fluorouracil; oxaliplatin; colon cancer cell lines; drug combination

For more than four decades, 5-fluorouracil (FU) has been the only drug offering acceptable efficacy in the chemotherapeutic management of colorectal cancer. More recently, the response rate of FU has been shown to be markedly improved by combination with folinic acid (FA) (Advanced Colorectal Cancer Meta-analysis Project, 1992).

There are currently new active anticancer drugs emerging in the area of colorectal cancer treatment. Two drugs are of particular interest: a new platinum derivative, oxaliplatin (LOHP) and a camptothecin analogue, CPT11. LOHP as a single agent, shows significant activity in colorectal cancer but, more interestingly, its association with FUFA has been demonstrated to be superior to FUFA in terms of antitumor efficacy in colorectal cancer (De Gramont et al, 2000). These clinical results confirm previous preclinical data indicating synergistic effects when combining these drugs (Raymond et al, 1997; Fischel et al, 1998). In the same way, CPT11 alone is active in colon cancer and, interestingly, in patients refractory to FU (Cunningham et al, 1998; Rougier et al, 1998). More recently, it has been shown that the CPT11-FUFA combination produced a higher response rate (Douillard et al, 2000; Saltz et al, 2000) and prolonged survival (Douillard et al, 2000) in comparison to FUFA in advanced colorectal cancer patients. As for the LOHP–FUFA association, these clinical results are in agreement with preclinical data showing synergistic interactions between CPT11 and FUFA (Guichard et al, 2000; Pavillard et al, 1998).

This background survey invites consideration of a possible triple combination involving CPT11, LOHP and FUFA. This association is particularly justified by the specific differences in the respective targets of drug action. The purpose of the present work was to compare different schedules for the CPT11–LOHP–FUFA combination. Experimental conditions were established so as to adopt a clinically relevant schedule and to take into account previous experimental results (Fischel et al, 1998). To this end, the LOHP–FUFA schedule was designed so as to reflect the widely used ‘de Gramont’ protocol in which a 2 h LOHP sequence precedes a 48 h FU exposure (De Gramont, 1997). SN38, the active metabolite of CPT 11, was introduced into this fixed LOHP–FUFA sequence thus giving rise to two different schedules in which SN38 was applied either before (schedule A) or after (schedule B) LOHP. SN38 was used because CPT11 is inactive per se and needs to be activated in the organism into SN38, the active drug (O’Reilly and Rowinski, 1996). The study was undertaken on two human colon cancer cell lines (WIDR and SW 620) that express spontaneous sensitivity to the tested drugs. Both WIDR and SW 620 cells were mutated for the *p53* gene. We took tumour cell lines with *p53* mutations because a majority of human colorectal cancer carry a *p53* mutational status (Kressner et al, 1999).

*Received 3 April 2000*
*Revised 27 September 2000*
*Accepted 14 October 2000*

*Correspondence to:* G Milano. E-mail: gerard.milano@nice.fnclcc.fr

### MATERIALS AND METHODS

### Chemicals

All of the chemicals including MTT, *l* ascorbic acid, and *dl* 5-methyltetrahydrofolate were obtained from Sigma Chemical Co (St Quentin Fallavier, France) and were of the highest purity available. Folic acid-free DMEM was obtained from Life Technologies, Inc (Paisley, Scotland). Regular DMEM and glutamine were obtained from Whittaker (Verviers, Belgium) and fetal bovine serum from Dutscher (Brumath, France). Penicillin and streptomycin were obtained from Merieux (Lyon, France). FU and LOHP were the pharmaceutical forms obtained from Roche (Neuilly-sur-Seine, France) and Sanofi Winthrop (Gentilly, France), respectively. FA (pure *l* FA) was the pharmaceutical form obtained from Wyeth-Lederle (Paris, France). SN 38 (7-ethyl-10-hydroxycamptothecin) was provided by Rhone Poulenc Rorer (Paris, France).

### Cell lines

Two colon cancer cell lines of human origin were used (Table 1). Cells were routinely cultured in DMEM supplemented with 10% fetal bovine serum, 2 mM glutamine, 50 000 U $l^{-1}$ penicillin and 80 µM streptomycin in a humidified incubator (Sanyo, Japan) at 37˚C with an atmosphere containing 8% $CO_2$. One week before experiments began, the cells were grown in a folate-controlled medium (folic acid-free DMEM supplemented with 40 nM of *dl* 5-methyltetrahydrofolate and 0.1 mM of *l* ascorbic acid) to simulate as closely as possible the physiological situation encountered in humans (Kones, 1990). The above folate-controlled medium was used throughout the experiments.

### Evaluation of cytotoxicity

Cells were seeded in 96-well microtitration plates (100 µl $well^{-1}$) to obtain exponential growth for the whole duration of the experiment (initial cell density was 3600 and 2500 cells $well^{-1}$ for SW620 and WIDR, respectively). 24 h later, cells were exposed to the drugs. We previously established that an optimal interaction was obtained between SN38 and FUFA when SN38 was applied before FUFA (Pavillard et al, 1998). In complement to these previously published data we made preliminary experiments so as to select the drug sequence to be definitively tested in the present study. We thus compared 5 different sequences on WIDR cells. Sequence I with SN 38 (24 h) then medium during 24 h and then LOHP (2 h) followed by FU (48 h); sequence II with SN 38 (24 h) followed by LOHP (2 h) and then FU (48 h); sequence III with LOHP (2 h) followed by SN 38 (24 h) and then FU (48 h); sequence IV with LOHP (2 h) followed by FU (24 h) and then SN 38 and FU together during 24 h and sequence V with LOHP (2 h) followed by FU (48 h) and then SN 38 (24 h). The decreasing order of cytotoxic efficacy was as follows, I and III > II > IV >> V as attested by the respective IC 50 values (mean  SD, $n$ = 3) for LOHP (µM), FU (µM) and SN 38 (µM) respectively:

Sequence I: 5.2 ± 1.2; 1.67 ± 0.38; 0.83 ± 0.19;

Sequence II: 11.4 ± 2.0; 3.65 ± 0.64; 1.8 ± 0.9;

Sequence III: 7.6 ± 1.8; 2.44 ± 0.58; 1.2 ± 0.28;

Sequence IV: 15.6 ± 4.1; 5.0  1.31; 2.5 ± 0.66 and

Sequence V: 22.1 ± 3.1; 7.1 ± 1.0; 3.5 ± 0.5.

Sequences I and III were thus kept for the definitive experiments; they correspond to schedule A and schedule B, respectively (Figure 1). Pure *l* FA was always tested at 10 µM and did not exhibit any effect on cell proliferation when tested alone. Concentration ranges were as follows: 3 $10^{-7}$ M < (LOHP) < 3 $10^{-3}$ M; 9 $10^{-11}$ M < (SN38) < 9 $10^{-7}$ M; 1.8 $10^{-7}$ M < (FU) < 1.8 $10^{-3}$ M. 11 concentrations were tested for each drug. When combined, the drugs were tested at a constant concentration ratio for a given cell line, the ratio being dictated by the drug sensitivity and close to the ratio of the IC50 of each drug (LOHP/SN38 ratios were 3320 and 6250 for SW620 and WIDR respectively, those of LOHP/FU were 1.66 and 3.12, respectively). Experimental conditions were tested in sextuplicate (6 wells of the 96-well plate per experimental condition), and experiments were performed at distance in triplicate. Growth inhibition was assessed by the MTT test (Carmichael et al, 1987) 120 h after the start of drug exposure. Results were expressed as the relative percentage of absorbance compared with controls without drug. The dose–effect curves were analysed on Graphad Software (Institute for Scientific Information, Philadelphia, PA).

The cytotoxic effects obtained with the different drug combinations were analysed according to the Chou and Talalay method (1984) on Calcusyn software (Biosoft, Cambridge, United Kingdom). For that purpose, FUFA was considered as a single drug. Interaction between the 2 drugs or the 3 drugs together was assessed by means of an automatically computed combination index. Combination indexes were determined at 50% and 75% cell lethality. Combination index is defined as follows:

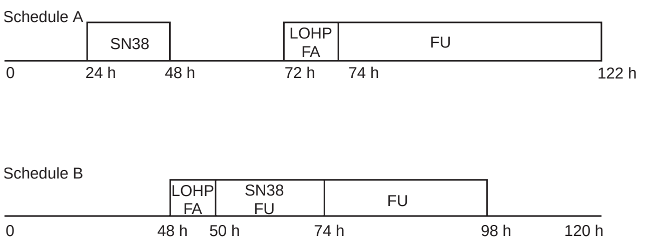

**Figure 1** Drug combinations tested on cell lines

**Table 1** Cell line characteristics

| Cell line | Origin[a] | P53 status[b] | FU IC50[c] (mean value, µM) | LOHP IC50[c] (mean value, µM) | SN38 IC50[c] (mean value, µM) |
|---|---|---|---|---|---|
| SW620 | ATCC (CCL 227) | mutated | 8.2 | 7.2 | 0.0036 |
| WIDR | EORTC | mutated | 5.2 | 19.6 | 0.0039 |

[a]ATCC, American Type Culture Collection; EORTC, European Organization for Research and Treatment of Cancer. [b]P53 mutations were determined by Dr P Laurent-Puig (INSERM U490, Paris). [c]IC50 means concentration inhibiting 50% of cell proliferation. See ‘Materials and Methods’ section for details on drug exposure conditions.

 *© 2001 Cancer Research Campaign*

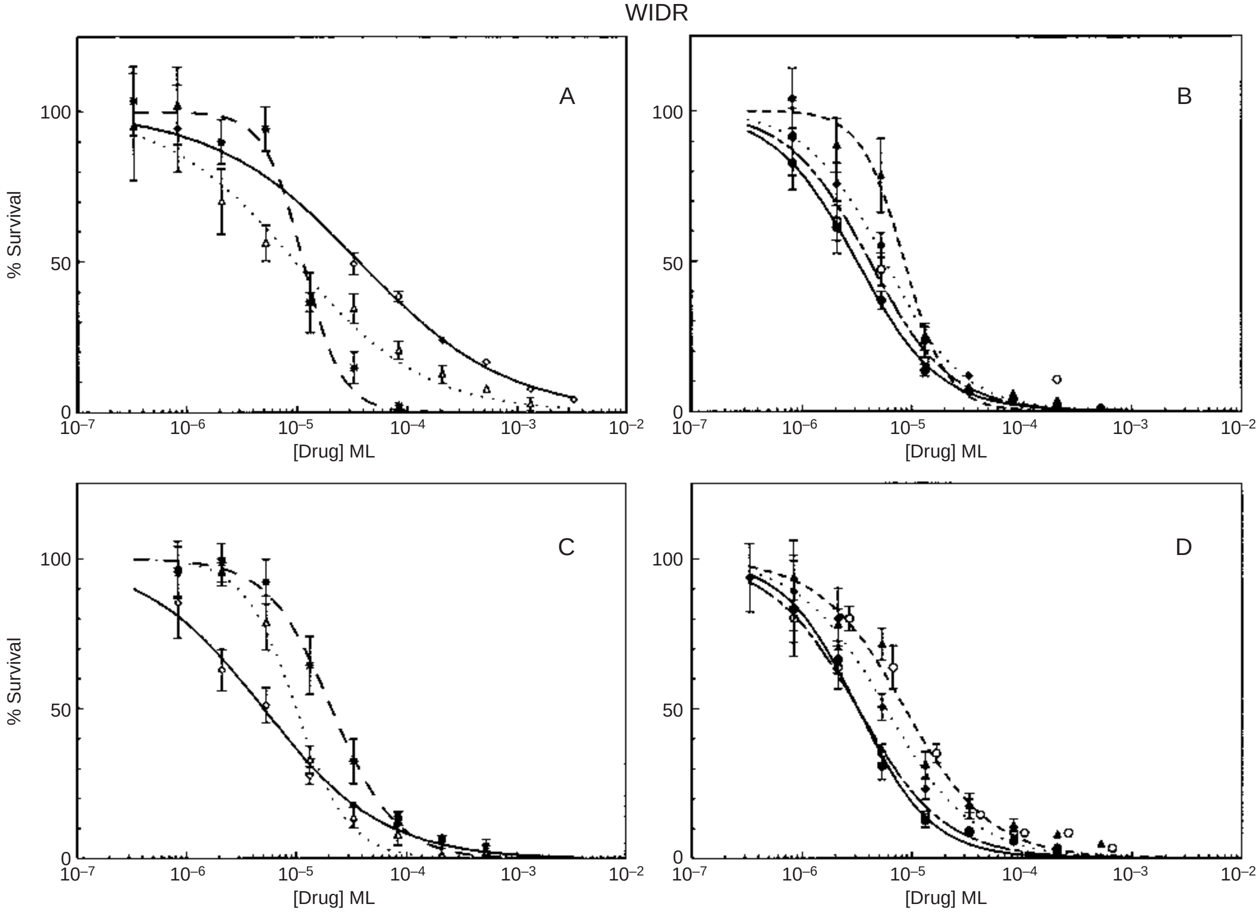

**Figure 2** In **A** and **C**, dose–effect curves of the different drugs tested alone (✻: SN38; △: FUFA ◇: LOHP); in **B** and **D**, dose–effect curves of the drugs tested in combination (○: LOHP – FUFA; ▲: FUFA – SN38; ◆: LOHP – SN38; ●: LOHP – FUFA – SN38). Figures 2A and 2B concern WIDR cell line exposed to schedule A and Figures 2C and 2D concern WIDR cell line exposed to schedule B. The horizontal axis directly expresses the drug concentration for LOHP (M/L). The tested SN38 concentration is obtained by dividing the reading concentration by 6250 and the tested FU concentration is obtained by dividing the reading concentration by 3.12

$$CI_{A+B} = \frac{D_{A/A+B}}{D_A} + \frac{D_{B/A+B}}{D_B} + \alpha \frac{D_{A/A+B} \times D_{B/A+B}}{D_A D_B}$$

with $CI_{A+B}$ = combination index for a fixed effect (F) for the combination of drug A and a drug B.

$D_{A/A+B}$ = concentration of drug A in the combination A + B giving an effect F.

$D_{B/A+B}$ = concentration of drug B in the combination A + B giving an effect F.

$D_A$ = concentration of drug A alone giving an effect F.

$D_B$ = concentration of drug B alone giving an effect F.

$\alpha$ = parameter with value 0 when A and B are mutually exclusive and 1 when A and B are mutually non-exclusive.

The combination index indicated synergism when smaller than 0.80, antagonism when greater than 1.20, and additive cytotoxic effects when located between 0.80 and 1.20.

### Statistics

Comparisons were performed on the whole cell line panel by means of nonparametric ANOVA matched for cell lines and experiments (Friedman test). Statistics were drawn up on SPSS software (Chicago, IL).

### RESULTS

Typical dose–effect curves for the different drug combinations are displayed in Figure 2 for WIDR and Figure 3 for SW620. In all cases, the conditions with all drugs applied together generated the concentration–response curves at the left extremity meaning that the best cytotoxic effects were obtained in these cases.

The combination indexes (CI) computed at 50% and 75% cell lethality are given in Table 2 for both cell lines. Based on these CI values, it appears that the LOHP–FUFA combination was globally synergistic, LOHP–SN38 either additive or antagonistic, FUFA–SN38 antagonistic, and the triple combination resulted in additive effects. Typical examples for CI/fractional effects curves are given in Figures 4–6.

The influence of the SN38 position in the triple combination was further analysed by comparing the CI (Wilcoxon test matched for cell lines, experiments and final cytotoxic effects). The observed CI were significantly different according to the SN38 position ($P = 0.03$), thus leading to a shift from a median value at 1.05 when

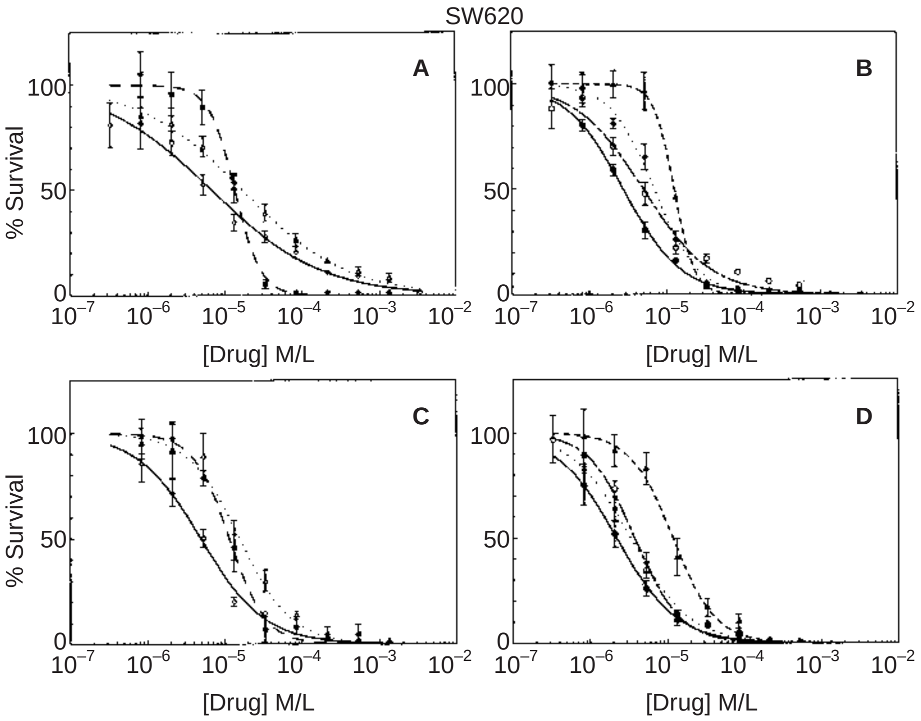

**Figure 3** In **A** and **C**, dose–effect curves of the different drugs tested alone (✻: SN38; △: FUFA; ◇: LOHP); in **B** and **D** dose–effect curves of the drugs tested in combination (○: LOHP – FUFA; ▲: FUFA – SN38; ◆: LOHP – SN38; ●: LOHP – FUFA – SN38). Figures 3A and 3B concern SW620 cell line exposed to schedule A and Figures 3C and 3D concern SW 620 cell line exposed to schedule B. The horizontal axis directly expresses the drug concentration for LOHP (M/L). The tested SN38 concentration is obtained by dividing the reading concentration by 3320 and the tested FU concentration is obtained by dividing the reading concentration by 1.66

**Table 2** Combination indexes for the different drug associations

| Drug combination | Sequence with SN38 | % effect | Combination indexes[a] | | |
|---|---|---|---|---|---|
| | | | WIDR (mean ± SD) | SW620 (mean ± SD) | General pattern |
| LOHP-FUFA | | 50 | 0.60 ± 0.20 | 0.85 ± 0.13 | Synergistic |
| | | 75 | 0.45 ± 0.31 | 0.70 ± 0.24 | |
| | | r ± SD[b] | 0.992 ± 0.004 | 0.982 ± 0.030 | |
| LOHP-SN38 | Schedule A (without FU) | 50 | 1.60 ± 0.16 | 1.45 ± 0.31 | Additive or antagonistic |
| | | 75 | 1.35 ± 0.50 | 1.13 ± 0.21 | |
| | | r ± SD | 0.993 ± 0.005 | 0.979 ± 0.026 | |
| | Schedule B (without FU) | 50 | 1.23 ± 0.13 | 1.00 ± 0.10 | |
| | | 75 | 1.20 ± 0.19 | 1.10 ± 0.18 | |
| | | r ± SD | 0.992 ± 0.004 | 0.991 ± 0.005 | |
| FUFA-SN38 | Schedule A (without LOHP) | 50 | 1.55 ± 0.21 | 1.43 ± 0.64 | Antagonistic |
| | | 75 | 1.28 ± 0.26 | 1.00 ± 0.45 | |
| | | r ± SD | 0.987 ± 0.007 | 0.986 ± 0.009 | |
| | Schedule B (without LOHP) | 50 | 2.22 ± 2.45 | 1.20 ± 0.61 | |
| | | 75 | 2.00 ± 1.12 | 1.70 ± 0.57 | |
| | | r ± SD | 0.978 ± 0.020 | 0.981 ± 0.017 | |
| SN38-LOHP-FUFA | Schedule A (all drugs) | 50 | 1.00 ± 0.36 | 0.93 ± 0.22 | Additive |
| | | 75 | 0.80 ± 0.13 | 0.78 ± 0.15 | |
| | | r ± SD | 0.992 ± 0.004 | 0.988 ± 0.014 | |
| | Schedule B (all drugs) | 50 | 1.43 ± 0.68 | 0.985 ± 0.30 | |
| | | 75 | 1.10 ± 0.13 | 1.10 ± 0.32 | |
| | | r ± SD | 0.991 ± 0.007 | 0.985 ± 0.007 | |

Schedule A corresponds to the sequence where SN 38 is applied first and schedule B corresponds to the sequence where SN 38 is applied after LOHP (see Figure 1 for details); [a]: Combination indexes (CI), computed according to the Chou and Talalay method (Calculsyn Software), indicated synergism when smaller than 0.80, antagonism when greater than 1.20, and nearly additivity between 0.80 and 1.20; [b]: r value is the coefficient of correlation for the fitting between CI values and fractional effects (5 to 7 experimental points between 0.2 and 1.0); SD means standard deviation. For the triple association (SN 38 – LOHP – FUFA) the CIs are computed by taking into account the dose-response curves of each individual drug.

 *© 2001 Cancer Research Campaign*

**Table 3** Relative contributions of each drug to the overall cytotoxicity of the triple association[a]

| | | SN38 contribution | FUFA contribution | LOHP contribution | Statistics[b] |
|---|---|---|---|---|---|
| Whatever the schedule | median | 1.12 | 1.59 | 2.42 | $P = 0.002$ |
| | 1st–3rd quartile | 0.95–1.58 | 1.12–2.62 | 1.47–3.88 | |
| Schedule A | median | 1.16 | 2.24 | 2.42 | $P = 0.002$ |
| | 1st–3rd quartile | 0.98–1.57 | 1.35–2.67 | 1.53–4.13 | |
| Schedule B | median | 1.12 | 1.23 | 2.46 | $P = 0.32$ |
| | 1st–3rd quartile | 0.90–1.65 | 1.03–1.88 | 1.06–3.88 | |

Data are averaged values by grouping the results of WIDR with those of SW 620. [a]The relative contribution of each drug was defined as the IC50 of the double association without that drug divided by the IC50 of the triple association (a ratio at 1 indicated no contribution at all). [b]Comparison of the relative contributions of SN38, FUFA and LOHP according to the Friedman test, paired on cell lines and experiments.

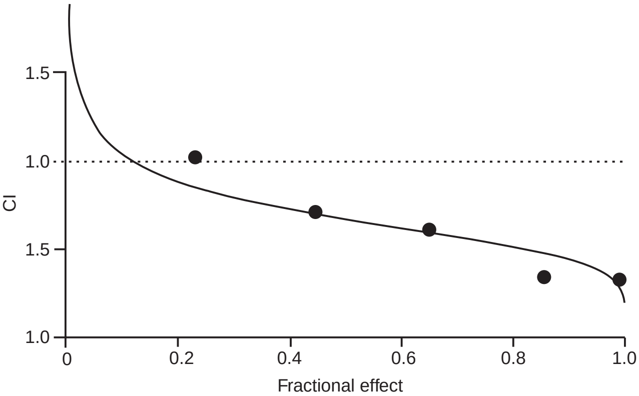

**Figure 4** Typical combination index/fractional effect curve derived from Chou and Talalay model: LOHP–FUFA, WIDR cells (globally synergistic)

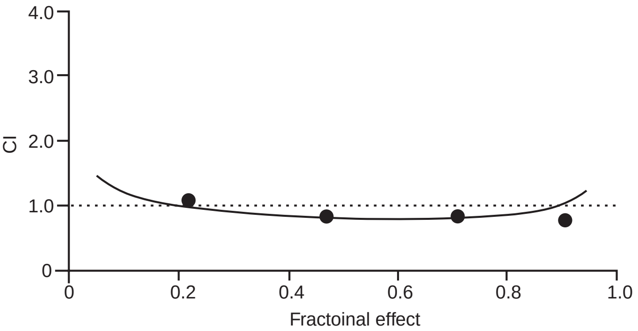

**Figure 5** Typical combination index/fractional effect curve derived from Chou and Talalay model: SN 38 – LOHP – FUFA, schedule A, SW 620 cells (globally additive)

SN38 was applied after LOHP (1st–3rd quartile 0.90–1.38, range 0.70–2.40) to a median value at 0.80 when SN38 was applied first (1st–3rd quartile 0.70–1.00, range 0.60–1.50). Comparison of the CI resulting from the triple association (additivity pattern) with the CI resulting from the LOHP–FUFA association (synergistic pattern) indicates that the presence of SN38 does not add to the cytotoxicity already confered by the LOHP–FUFA combination (Table 2).

The relative contribution of each drug to the overall cytotoxicity of the triple combination was then analysed by computing the ratio defined as the drug concentration giving 50% of cell lethality (IC50) of the double association without that drug divided by the IC50 of the triple association (the higher the ratio, the greater

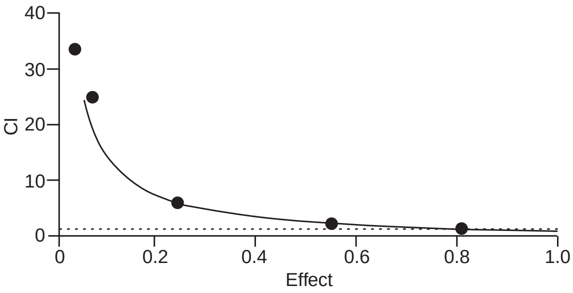

**Figure 6** Typical combination index/fractional effect curve derived from Chou and Talalay model: SN 38 – LOHP – FUFA, schedule B, WIDR cells (globally antagonist)

the contribution of that given drug). Table 3 gives the values of relative contributions for SN38, FUFA and LOHP. Statistical analysis (Friedman test paired on cell lines, experiments and schedules) indicated that relative contributions of each of the 3 drugs are significantly different ($P = 0.002$, first line Table 3). Whatever the SN38 position, the greater contribution to the overall cytotoxicity of the triple combination comes from LOHP (median relative contribution = 2.4) and the smallest comes from SN38 (median relative contribution = 1.1). This analysis confirms that SN38 brings a relatively modest contribution to the cytotoxicity of the triple association. Interestingly, when considering schedules A and B separately, SN38 and LOHP relative contributions remained similar whereas the relative contribution of FUFA improved considerably when SN38 was applied first (Table 3).

Of note, when considering the results obtained from both cell lines (Figures 2 and 3, Table 2) it can be observed that a close agreement does exist between them.

### DISCUSSION

Given the recent clinical data in gastrointestinal cancer showing the very promising antitumour effects produced by combinations of LOHP and FUFA (De Gramont et al, 2000) and of CPT 11 and FUFA (Douillard et al, 2000), it is likely that the triple association between LOHP, CPT 11 and FUFA will soon be tested in cancer patients. Since each drug has its own significant toxicity, it is important to learn, at an experimental stage, what type of interaction might result from the combined effects of these three drugs. Answering this question was the central goal of the present study. In order to minimize the inevitable discrepancy between conclusions at the bench and clinical applications at the bedside, the drug combinations were tested by applying clinically compatible conditions. Most similar in vitro studies are often based on the use of a single tumour cell line. In the present work two p53 mutated human colon cancer cell lines were investigated and, interestingly, results globally concur fairly well between cell lines (Tables 2 and 4). This fact strengthens the impact of the present observations.

Analysis of the double association LOHP–FUFA showed a majority of synergistic interactions (Table 2). This corroborates previous results by others (Raymond et al, 1998) and us (Fischel et al, 1998). Such synergism could be related to the previously demonstrated reduced folate pool expansion under the effects of platinum derivatives (Scanlon et al, 1986; Shirasaka et al, 1993). In addition, recent pharmacokinetic data have suggested that LOHP can inhibit dihydropyrimidine dehydrogenase (DPD) which is the rate-controlling enzyme of FU catabolism (Gamelin et al, 1997), and that this DPD inhibition may enhance FUFA cytotoxicity (Milano and Etienne, 1994).

When examining the effects of the FUFA–SN38 combination, antagonistic effects were observed (Table 2). This observation differs from previous results showing synergism when applying SN38 before FUFA (Pavillard et al, 1998). A reason for this discrepency may lie in the different tested schedules, SN38–FUFA combination having been previously tested as two 48 h consecutive sequence for each drug, whereas in the present study a 24 h sequence was applied for SN38 and a 48 h sequence for FU.

The combination of LOHP and CPT11 was tested in early clinical investigations (Wasserman et al, 1999; Scheithauer et al, 1999). Interactions between the two drugs have been studied at pharmacokinetic level without evidence of noticeable modifications in the pharmacokinetics of each drug (Lokiec et al, 1997). Present data indicate that the association of SN38 and LOHP does not produce synergistic effects but, on the contrary, triggers mild antagonism (when SN38 is applied first) or additivity (when SN38 is applied after LOHP, Table 2). The present results differ from that recently obtained on the HT 29 colon cancer cell line (Zeghari-Squalli et al, 1999); the authors found a synergy when LOHP and SN38 were combined, SN 38 being applied with LOHP or before and after LOHP. The discrepency between the present results and those reported by Zeghari-Squalli and coworkers (1999) may be explained by the HT 29 cell line used by these later authors which is 100 fold less sensitive to SN38 than the cell lines investigated in the present study; another difference is the long exposure time to LOHP (24 h) which was applied by these authors as compared to the conditions of present study (LOHP, 2 h). In addition, pharmacological interactions may exist between these two drugs since irinotecan-related cholinergic syndrome has been reported to be induced by the coadministration of oxaliplatin (Valencak et al, 1998).

When SN38, LOHP and FUFA were combined, the position of SN38 had a significant influence on the CI, leading to a shift from additivity-antagonism when SN38 was applied after LOHP (schedule B), towards additivity-synergism when SN38 was applied first (schedule A, Table 2). Importantly, examination of the relative contributions of each of the 3 drugs in the resulting global cytotoxicity reveals that the contribution of FUFA was clearly two-fold greater in schedule A (median 2.24) as compared with schedule B (median 1.23), whereas the relative contributions of SN38 and LOHP were not modified (Table 3). Moreover, additional cytometry analyses performed in the present study demonstrated that SN38 significantly induced cell recruitment in the S-G2-M phases 24 h and 48 h after SN38 exposure (unshown data). Also, the impact of camptothecins on the cell cycle was previously reported by others (Goldwasser et al, 1996) and ourselves (Pavillard et al, 1998). Since FU acts preferentially on cells entering the S phase, all together these data indicate that the SN38 position significantly influences the cytotoxicity of FUFA: in schedule A, FU was applied 26 h after the end of SN38 exposure, at the time of S phase recruitment, thus leading to greater FU cytotoxic effects as compared to schedule B.

Importantly, this study clearly demonstrates that the drug which makes the greatest contribution in the triple combination is LOHP (median relative contribution around 2.4) in contrast to SN38 which, in comparison, brings a relatively modest contribution (median relative contribution around 1.1, Table 3). The clinical application of the ternary combination considered in the present study may lead to a combined toxicity in treated patients. As recently stressed by Ratain (1999), original associations of drugs should be first tested at experimental level before clinical trials are begun. The present results may help to objectively discuss the rationale of future clinical trials combining CPT11, LOHP and FU.

### REFERENCES

Advanced Colorectal Cancer Meta-analysis Project (1992) Modulation of fluorouracil by leucovorin in patients with advanced colorectal cancer: evidence in terms of response rate. *J Clin Oncol* **10**: 896–903

Baisch H (1975) Analysis of PCP-data to determine the fraction of cells in the various phases of cell cycle. *Rad Environ Biophys* **12**: 31–39

Carmichael J, De Graff WG, Gazdar AF, Minna JD and Mitchell JB (1987) Evaluation of a tetrazolium-based semiautomated colorimetric assay: assessment of chemosensitivity testing. *Cancer Res* **47**: 936–940

Chou T and Talalay P (1984). Quantitative analysis of dose-effects relationships: the combined effects of multiple drugs or enzyme inhibitors. *Adv Enzyme Regul* **22**: 27–55

Cunningham D, Pyrhönen S., James RD, Lunt CJA, Hickish TF, Heikkila R, Johannesen TB, Starkhammer H, Topham CA, Award L, Jacques C and Herait P (1998). Randomized trial of irinotecan plus supportive care versus supportive care alone after fluorouracil failure for patients with metastatic colorectal cancer. *Lancet* **352**: 1413–18

De Gramont A, Vignoud J and Tournigand C (1997). Oxaliplatin with high-dose leucovorin and 5 fluorouracil 48 hour continuous infusion in pretreated metastastic colorectal cancer. *Eur J Cancer* **33**: 214–219

De Gramont A, Figer A, Seymour M, Homerin M, Hmissi A, Cassidy J, Boni C, Cortes-Funes H, Cervantes A, Freyer G, Papamichael D, le Bail N, Louret C, Hendler D, de Brand F, Wilson C, Morran F and Bonetti A (2000) Leucovorin and fluorouracil with or without oxaliplatin as first-line treatment in advanced colorectal cancer. *J Clin Oncol* **18**: 2938–2947

Dauillard JY, Cunningham D, Roth AD, Navarro M, James RD, Karasek P, Jandi KP, Ireson T, Carmichael J, Alakl M, Gruia G, Awad L and Rougier P (2000) Irinotecan combined with fluorouracil compared with fluorouracil alone as first-line treatment for metastatic colorectal cancer: a multicentre randomised trial. *Lancet* **355**: 1041–1047

Fischel JL, Etienne MC, Formento P and Milano G (1998). Search for the optimal schedule for the oxaliplatin/5 fluorouracil association modulated or not by folinic acid: preclinical data. *Clin Cancer Res* **4**: 2529–2535

Gamelin E, Boisdnon-Cell M, Allain P, Tureau A, Brienza S, Krikrian A, Cvitkovic E and Larra F (1997). Pharmacokinetic interference between oxaliplatin (LOHP) and fluorouracil (5-FU) delayed 5-FU metabolism + inhibition by LOHP. *Proc Am Soc Clin Oncol* **38**: 223

Goldwasser F, Shimizu T, Jackman J, Hoki Y, O'Connor PM, Kohn KW and Pommier Y (1996) Correlations between S and G2 arrest and the cytotoxicity of camptothecin in human colon carcinoma cells. *Cancer Res* **56**: 4430–4437

Guichard S, Cussac D, Hennebelle I, Bugat R and Camal P (1997) Sequence-dependent activity of the irinotecan-5Fu combination in human colon-cancer model HT-2g in vitro and in vitro. *Int J Cancer* **73**: 729–734

Houghton JA, Schmidt C and Houghton PJ (1982). The effect of derivatives of folic acid on the fluorodeoxyuridylate-thymidylate synthase covalent complex in human colon xenografts. *Eur J Cancer Clin Oncol* **18**: 347–351

© 2001 Cancer Research Campaign

Kones R (1990) Folic acid. An update with new recommended daily allowances. *South Med J* **83**: 1454–1458

Kressner U, Inganas M, Byding S, Blikstad I, Pahlman L, Glimelius B and Lindmark G (1999). Prognostic value of *p53* genetic changes in colorectal cancer. *J Clin Oncol* **17**: 593–599

Lokiec F, Wasserman E and Santori J. et al (1997). Pharmacokinetics of the irinotecan/oxaliplatin combination: preliminary data of an ongoing phase I trial. *Proc Am Assoc Cancer Res* **38**: 76

Milano G and Etienne MC (1994). Potential importance of dihydropyrimidine dehydrogenase (DPD) in cancer chemotherapy. *Pharmacogenetics*, **4**: 301–306

O'Reilly S and Rowinsky ER (1996). The clinical status of irinotecan (CPT 11), a novel water-soluble camptothecin analogue. *Critic Rev Oncol Hematol* **24**: 47–70

Pavillard V, Formento P, Rostagno P, Formento JL, Fischel JL, Francoual M, Etienne MC and Milano G (1998). Combination of irinotecan (CPT 11) and 5-fluorouracil with an analysis of cellular determinants of drug activity. *Biochem Pharmacol* **56**: 1315–1322

Ratain MJ (1999) Drug combinations: dangerous liaisons or great expectations? *Annals Oncol* **10**: 375–376

Raymond E, Buquet-Fagot C, Djelloul S, Mester J, Cvitkovic E, Allain P, Louvet C and Gespach C (1997) Antitumor activity of oxaliplatin in combination with 5-fluorouracil and the thymidylate synthase inhibitor AG 337 in human colon, breast and ovarian cancers. *Anticancer Drugs* **8**: 876–885

Raymond E, Chaney SG, Taamma A and Cvitkovic E (1998) Oxaliplatin: a review of preclinical and clinical studies. *Annals Oncol* **9**: 1053–1071

Rougier P, Van Cutsem E, Bajetta E, Niederle N, Possinger K, Labianca R, Navarro M, Morant R, Bleiberg H, Wils J, Award L, Herait P and Jacques C (1998) Randomized trial of irinotecan versus fluorouracil by continuous infusion after fluororacil failure in patients with metastatic colorectal cancer. *Lancet* **352**: 1407–1412

Saltz LB, Cox JV, Blanke C, Rosen LS, Fehrembacher L, Moore MJ, Maroun JA, Ackland SP, Locker PK, Pirotta N, Elfring GL and Miller LL (2000) Irinotecan plus fluorouracil and leucovorin in metastatic colorectal cancer. Irinotecan study group. *N Engl J Med* **343**: 905–914

Scanlon KJ, Newman EM, Lu Y and Priest DG (1986) Biochemical basis of cisplatin and 5-fluorouracil synergism in human ovarian carcinoma cells. *Proc Natl Acad Sci* USA, **83**: 8923–8925

Scheithauer W, Kornek GV, Raderer M, Valencak J, Weinlander G, Hejna M Haider K, Kwasny and W Depisch D (1999) Combined irinotecan and oxaliplatin plus granulocyte colong-stimulating factor in patients with advanced fluororpyrimdine/leucovorinpretreated colorectal cancer. *J Clin Oncol* **17**: 902–906

Shirasaka T, Shimamoto Y, Ohshimo H, Saito H and Fukushima M (1993) Metabolic basis of the synergistic antitumor activities of 5-fluorouracil and cisplatin in rodent models *in vivo. Cancer Chemother Pharmacol* **32**: 167–172

Valencak J, Raderer M, Kornek GV, Henja MLH and Scheithauer W (1998) Irinotecanrelated cholinergic syndrome induced by coadministration of oxaliplatin. *J Natl Cancer Inst* **90**: 160

Vindelov LL, Christensen ID and Nissen NI (1983) A detergent trypsin method for the preparation of nuclei for flow cytometry DNA analysis. *Cytometry* **3**: 323–327

Wasserman E, Cuvier C, Lokiec F, Goldwasser F, Kalla S, Mery-Mignard D, Ouldkaci M, Besmaine A, Dupont-André G, Mahjovbi M, Marty M, Misset JL and Critkorie E (1999) Combination of oxaliplatin plus irinotecan in patients with gastrointestinal tumors: results of two independent phase I studies with pharmacokinetics *J Clin Oncol* **17**: 1751–1759

Zeghari – Squalli N, Raymond E, Cvitkovic E and Goldwasser F (1999) Cellular pharmacology of the combination of the DNA topoisomerase I inhibitor SN – 38 and diaminocyclohexane platinum derivative oxaliplatin. *Clin Cancer Res* **5**: 1189–1196

*© 2001 Cancer Research Campaign*
